# Metabolismo óseo en niños aragoneses con normopeso y niños con sobrepeso/obesidad

**DOI:** 10.1515/almed-2023-0065

**Published:** 2024-01-01

**Authors:** José Cuenca Alcocel, Lorena Villalba-Heredia, Inés Martínez Redondo, Clara Berrozpe-Villabona, José Antonio Casajús, José Miguel Arbonés-Mainar, Pilar Calmarza

**Affiliations:** Servicio de Bioquímica Clínica, Hospital Obispo Polanco, Teruel, España; GENUD Research Group, Universidad de Zaragoza, Zaragoza, España; Servicio de Pediatría, Hospital Universitario Miguel Servet, Zaragoza, España; Servicio de Medicina Preventiva, Hospital Universitario Miguel Servet, Zaragoza, España; GENUD (Growth, Exercise, Nutrition and Development) Research Group, Universidad de Zaragoza, Instituto de Investigación Sanitaria Aragón (IIS Aragón) Zaragoza, España; Centro de Investigación Biomédica en Red de Fisiopatología de la Obesidad y Nutrición (CIBEROBN), Instituto de Salud Carlos III Madrid, España; Departamento de Fisiatría y Enfermería, Facultad de Ciencias de la Salud y el Deporte, Universidad de Zaragoza Zaragoza, España; Adipocyte and Fat Biology Laboratory (AdipoFat), Unidad de Investigación Transversal, Hospital Universitario Miguel Servet, Instituto de Investigación Sanitaria (IIS), Zaragoza, Aragón, España; Instituto Aragonés de Ciencias de la Salud (IACS), Zaragoza, España; CIBER Fisiopatología Obesidad y Nutrición (CIBERObn), Instituto Salud Carlos III, Madrid, España; Servicio de Bioquímica Clínica, Hospital Universitario Miguel Servet, Zaragoza, España; Centro de Investigación en Red en Enfermedades Cardiovasculares (CIBERCV), Universidad de Zaragoza, Instituto de Investigación Sanitaria Aragón (IIS Aragón) Zaragoza, España; Miembro de las Comisiones de Estrés Oxidativo y Lipoproteínas y Enfermedades vasculares de la SEQC-ML, Servicio de Bioquímica Clínica, Hospital Universitario Miguel Servet Zaragoza, España

**Keywords:** metabolismo óseo, sobrepeso/obesidad, marcadores recambio óseo, niños, correlación

## Abstract

**Objetivos:**

En la infancia y adolescencia se produce un aumento de masa ósea, hasta alcanzar un pico máximo, determinante para la salud ósea. Los marcadores óseos evalúan los procesos de formación-resorción ósea. Sin embargo, los estudios sobre la influencia de la obesidad en los marcadores de recambio óseo en esta edad, son escasos y los resultados contradictorios. El objetivo de nuestro estudio fue evaluar si el sobrepeso/obesidad influían en el metabolismo óseo.

**Métodos:**

Se compararon parámetros relacionados con el metabolismo óseo, en 45 niños y niñas normopeso (controles) y en un grupo de 61 niños y niñas con sobrepeso/obesidad (casos), procedentes del estudio Exergames (Universidad de Zaragoza), de edades comprendidas, todos ellos, entre 8 y 12 años.

**Resultados:**

La concentración de fósforo y la de IGFBP-3 fueron superiores en los niños con sobrepeso/obesidad, respecto a la de los niños normopeso, (p=0,042) y (p=0,042), respectivamente. Las concentraciones de BAP, osteocalcina, magnesio, vitamina D e IGF-I fueron más bajas en el grupo de los niños con sobrepeso/obesidad y la de calcio más elevada, pero las diferencias no fueron estadísticamente significativas. Existe correlación negativa (r=−0,193) (p=0,049) entre BAP e IMC.

**Conclusiones:**

En los niños con sobrepeso/obesidad pese a que no se llegó a alcanzar significación estadística, la concentración de BAP y osteocalcina fue inferior a la de los niños normopeso, lo cual junto con la correlación negativa de BAP respecto al IMC encontrada, puede indicar que ya en edades tan tempranas el sobrepeso/obesidad puede afectar a la salud ósea.

## Introducción

La obesidad en la edad infantil afecta tanto al inicio del desarrollo como a la maduración puberal y puede tener un impacto en la pubertad temprana y en el desarrollo sexual, asociándose con una edad más temprana de inicio de la pubertad y un adelanto en el desarrollo de las características sexuales secundarias en ambos sexos [1].

La prevalencia de la obesidad en la infancia y/o adolescencia ha aumentado en los países europeos [2], hasta llegar a representar actualmente un importante problema de salud pública mundial y un riesgo para el desarrollo futuro de enfermedades cardiovasculares y diabetes mellitus de tipo 2 [3]. Según el estudio ALADINO 2019 [4] y algunos otros estudios [5, 6], pese a que, al parecer, en los últimos años la prevalencia de la obesidad y el sobrepeso en la edad infantil se ha estancado, ésta se ha situado en índices muy altos, en torno al 20 %, para edades comprendidas entre los 6 y los 12 años.

El hueso es un tejido metabólicamente activo, en el que los procesos de formación y resorción ósea tienen lugar de forma simultánea y en lugares diferentes [7]. Durante la infancia y la adolescencia, el metabolismo óseo está incrementado y adaptado a las necesidades de crecimiento del esqueleto, produciéndose un aumento de la masa ósea hasta alcanzar un pico máximo conocido como “masa ósea pico” [8]. El máximo de masa ósea alcanzada es un factor determinante muy importante para su futura salud ósea. La edad en la que se alcanza el pico de masa ósea varía en función de diversos factores, entre los cuales se encuentran el sexo y la genética. En general, se estima que el pico de masa ósea se alcanza aproximadamente entre los 18–23 años de edad [8], de forma que una baja masa ósea supone un riesgo aumentado del número de fracturas y predispone al desarrollo de enfermedades óseas como la osteoporosis [9].

Sin embargo, los estudios que evalúan el metabolismo óseo en niños obesos y con sobrepeso son escasos, encontrándose en algunos de los mismos, resultados contradictorios. Así, en algunos de estos estudios se evidenció que los niños y adolescentes con sobrepeso/obesidad presentaban una masa ósea disminuida [10], mientras que en otros los niños/as con sobrepeso/obesidad presentaban densidad mineral ósea (DMO) superior a la de los niños normopeso [11], no conociéndose, en este caso, el impacto sobre la salud ósea en la edad adulta.

Los marcadores de recambio óseo son una serie de sustancias que se producen durante el proceso de remodelado óseo. Miden los productos generados durante la formación y degradación de la matriz ósea y pueden determinarse en suero y orina. Su análisis repetido, en intervalos cortos de tiempo, permite una evaluación seriada del recambio óseo, ofreciendo una información dinámica y real sobre el esqueleto. En los niños es necesario conocer la velocidad de crecimiento y el desarrollo puberal para interpretar correctamente los resultados [12]. Algunos de los marcadores de remodelado óseo, más utilizados, incluyen la osteocalcina y la fosfatasa alcalina ósea (BAP), que evalúan el proceso de formación ósea, y entre los marcadores que evalúan el proceso de resorción ósea se encuentran el β-CrossLaps (β-CTx) o el telopéptido C-terminal del colágeno tipo I (ICTP).

En cuanto a estos marcadores de remodelado óseo, en los niños con sobrepeso/obesidad, se han observado, asimismo, muchas discrepancias, mientras que en algunos estudios se observó que los niños con obesidad presentaban valores de marcadores óseos más bajos que los niños normopeso [13, 14], en otros, los niños obesos presentaban concentraciones de marcadores de remodelado óseo similares a las de los niños normopeso [15].

También se pudo comprobar, en algunos estudios, que la obesidad en los niños estaba relacionada con diversos marcadores de inflamación como la interleucina-6 (IL-6), la proteína C reactiva (PCR) y el factor de necrosis tumoral-α (TNF-α), entre los cuales había algunos que disminuían la DMO, mientras que otros mejoraban la acumulación de minerales óseos [16].

El objetivo de este estudio ha sido evaluar, en niños de 8–12 años con normopeso y niños con obesidad o sobrepeso procedentes, estos últimos, de un estudio llevado a cabo en la Universidad de Zaragoza (Exergames), parámetros del metabolismo óseo (marcadores de remodelado óseo: osteocalcina y fosfatasa alcalina ósea (BAP), vitamina D, calcio, magnesio, proteína transportadora 3 del factor de crecimiento similar a la insulina (IGFBP-3) y somatomedina C (IGF-I) y evaluar si el sobrepeso/obesidad puede influir en estos parámetros.

## Materiales y métodos

Se trata de un estudio observacional de casos y controles que fue realizado en Zaragoza, Aragón, España. Para el grupo de los controles se partía de un grupo de 59 niños y niñas normopeso que iban a ser sometidos a una cirugía de importancia menor (criptorquidia, fimosis, traumatológica, entre otros), en el cual, tras revisión de las historias clínicas, se excluyeron aquellos que presentaban alguna patología, así como los que presentaban sobrepeso u obesidad, empleando los criterios de clasificación de la *International Obesity Task Force* (IOTF) [17].

Tras aplicar estos criterios el grupo de niños normopeso, quedó constituido por 45 niños.

El grupo de casos estaba formado por 61 niños y niñas con sobrepeso u obesidad (calculado mediante el IMC y siguiendo también los puntos de corte definidos por Cole et al. [17], ajustados por edad y talla, con un equivalente de IMC 25 y 30 kg/m^2^, sobrepeso y obesidad, respectivamente).

Dichos niños procedían del estudio Exergames, llevado a cabo en la Universidad de Zaragoza.

**Figura 1:**
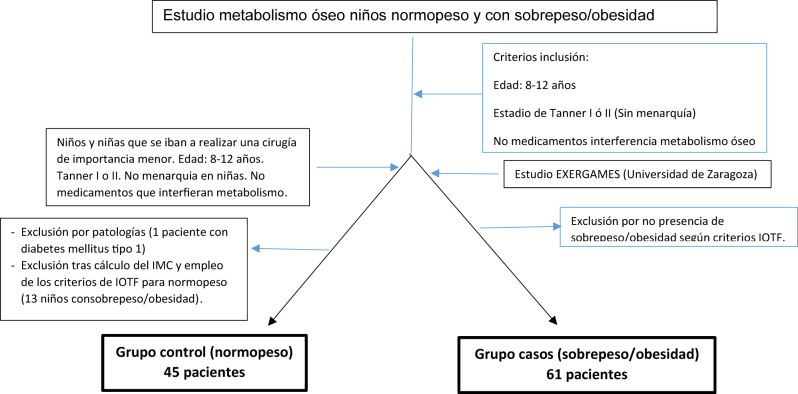
Selección del grupo niños normopeso (grupo control) y niños con sobrepeso/obesidad (grupo de casos).

En ambos casos se seleccionaron niños de edades comprendidas entre los 8 y 12 años, procedentes de la Comunidad Autónoma de Aragón, que no hubieran iniciado el desarrollo puberal, ni la menarquia en las niñas (estadios I y II de Tanner), que no tomaran suplementos de vitamina D, así como que no presentaran patologías o tratamientos que pudieran influir en los parámetros del estudio (enfermedades óseas, metabólicas, crónicas, infección aguda, anorexia nerviosa). La selección de los grupos de estudio se esquematiza en la Figura 1.

En primer lugar, se informó a los padres del estudio a realizar, presentándoles, toda la información por escrito, así como el documento del consentimiento informado, el cual fue firmado por todos ellos. A continuación, se realizó una breve encuesta sobre datos epidemiológicos y clínicos (Material Suplementario, Anexo 1), medición de las variables antropométricas -peso, talla e índice de masa corporal (IMC)- y exploración física. Posteriormente, se les realizó una analítica en ayunas, donde se determinaron los parámetros de interés para la evaluación del metabolismo óseo.

Las muestras de sangre se extrajeron a primera hora de la mañana, tras ayuno nocturno, en tubos con gel separador y se analizaron en suero los siguientes parámetros: calcio, fósforo y magnesio, los cuales se determinaron mediante técnicas espectrofotométricas en un Autoanalizador AU 5800 (Beckmann Coulter Miami, FL, EE.UU.). Osteocalcina, IGFBP-3 e IGF-I se determinaron mediante un inmunoensayo con detección por electroquimioluiniscencia, totalmente automatizado en un Analizador Cobas e411 (Roche Diagnostics, España). La concentración de BAP se determinó mediante un ensayo ELISA manual, con posterior lectura espectrofotométrica y la concentración de vitamina D, mediante inmunoanálisis automatizado en el Analizador Architect i1000SR (Abbott Diagnostics, EE.UU.).

Este estudio cumple con todas las regulaciones nacionales, políticas institucionales y principios éticos de la Declaración de Helsinki, y fue aprobado por el Comité de Ética en Investigación de la Comunidad Autónoma de Aragón (CEICA).

Para el análisis estadístico se utilizó el programa IBM SPSS Statistics versión 26.0.

En primer lugar, tanto para los parámetros antropométricos como para los parámetros bioquímicos, se empleó el test de Kolmogorov-Smirnov, con modificación de Lilliefors (KSL) para estudiar la distribución de las variables cuantitativas. En el caso de que siguieran una distribución normal (KSL, p>0,05) se emplearon para su descripción la media y la desviación estándar y en el caso de que las variables cuantitativas no siguieran una distribución normal (KSL, p≤0,05), se emplearon para su descripción la mediana y el rango intercuartílico.

Para la comparación entre los dos grupos, de los parámetros antropométricos y bioquímicos se utilizó la prueba t-Student o el test de Welch, cuando se trataba de una distribución normal, dependiendo respectivamente de si las varianzas en ambos grupos eran homogéneas o no, y el test U-Mann Whitney cuando se trataba de una distribución no normal.

Se realizó, también, un análisis estadístico similar, utilizando los test citados anteriormente, para estudiar la idoneidad de los dos grupos de estudio, en cuanto a edad, talla e IMC, comprobando si eran comparables en cuanto a estos parámetros.

Para estudiar la correlación de los parámetros bioquímicos con el IMC, la edad y el sexo, primero se aplicó el test de KSL, a la totalidad de la muestra, para comprobar la normalidad de las variables, utilizando posteriormente el coeficiente de correlación de Pearson, cuando las variables seguían una distribución normal, el de Spearman cuando no la seguían y el de Tau-b de Kendall cuando la variable era dicotómica (como en el caso del sexo). El nivel de significación estadística para todos los test estadísticos empleados se estableció a partir de un valor p≤0,05.

## Resultados

Los datos antropométricos de los niños de cada uno de los grupos, así como los resultados más significativos de la encuesta realizada se muestran en la Tabla 1. En cuanto a la edad no se encontraron diferencias estadísticamente significativas entre ambos grupos y en cuanto a la proporción de niños y niñas, los resultados también fueron comparables entre ambos grupos (55,7 % de niños en el grupo de casos frente a 64,4 % en el grupo de niños control, p=0,064). El número de fracturas óseas presentadas fue también similar en ambos casos, presentando únicamente, uno de los niños del grupo de casos, fracturas de repetición (dos fracturas de localización diferente). La existencia de infecciones de repetición fue, asimismo, similar en ambos grupos.

**Tabla 1:** Parámetros antropométricos y más significativos de la encuesta realizada en niños con normopeso y niños con sobrepeso/obesidad.

	Sobrepeso/Obesidad (n=61, 34 niños y 27 niñas)			Normopeso (n=45, 29 niños y 16 niñas)			Significación estadística (test)	Valor p test de Levene
	Total	Límites	Pruebas de normalidad^c^	Total	Límites	Pruebas de normalidad^c^		
Edad, años	10,1 ± 0,9^a^	(8,4–12,2)	0,200	10,1 ± 1,1^a^	(8,4–12,0)	0,200	0,912 (Welch)	0,014
Peso, kg	55,4 (14,8)^b^	(33,4–89,1)	0,034	33,0 ± 8,2^b^	(22,0–42,0)	0,200	<0,001 (U Mann-Whitney)	–
Talla, cm	145±8^a^	(129–161)	0,200	138±9^a^	(119–155)	0,200	<0,001 (t-Student)	0,521
IMC	25,8 (4,0)^b^	(20,1–36,0)	0,200	17,1 ± 2,4^b^	(13,7–19,9)	0,023	<0,001 (U de Mann-Whitney)	–

	**Mediana**	**Límites**		**Mediana**	**Límites**			

Z-score peso	0,01	(-1,91–2,94)	–	0,13	(-1,91–1,80)	–	–	–
Z-score talla	−0,11	(-2,04–1,92)	–	0,09	(-2,24–1,96)	–	–	–
Z-score IMC	−0,02	(-1,75–3,01)	–	0,19	(-2,01–1,95)	–	–	–

	**Total**	**IC 95 %**		**Total**	**IC 95 %**			

Fracturas (% pacientes)	18,0	(9,4–30,0)	–	11,1	(3,7–24,1)	–	0,325 (Chi-cuadrado Pearson)	–
Infecciones de repetición (% pacientes)	52,5	(39,3–65,4)	–	45,5	(30,4–61,2)	–	0,479 (Chi-cuadrado Pearson)	–

^a^Media ± desviación estándar; ^b^mediana (rango intercuartílico); ^c^prueba de Kolmogorov-Smirnov con modificación de Lillefors.

Los resultados analíticos de los parámetros estudiados, así como los resultados obtenidos tras la aplicación de los distintos test estadísticos aplicados, se muestran en la Tabla 2.

**Tabla 2:** Tipo de distribución y estudio comparativo parámetros bioquímicos niños sobrepeso/obesidad y niños normopeso.

Parámetros	Sobrepeso/Obesidad		Normopeso		Significación estadística	Valor p test de Levene
	Valor	Pruebas de normalidad^c^	Valor	Pruebas de normalidad^c^		
BAP, U/L	124,7 ± 33,0 (116,2–133,1)^a^	0.200	134,0 ± 32,2 (124,2–143,8)^a^	0,200	0,151 (t-Student)	0,467
Osteocalcina, ng/mL	84,4; 37,2 (67,4–104,6)^b^	0,045	85,0; 36,8 (72,7–109,5)^b^	0,007	0,572 (U de Mann-Whitney)	–
Calcio, mg/dL	10,00; 0,5 (9,80–10,30)^b^	0,193	9,90; 0,5 (9,70–10,20)^b^	0,135	0,251 (U de Mann-Whitney)	–
Fósforo, mg/dL	5,04; 0,69 (4,61–5,30)^b^	0,200	4,90; 0,60 (4,50–5,10)^b^	0,002	**0,042** (U de Mann-Whitney)	–
Magnesio, mg/dL	2,00; 0,25 (1,95–2,20)^b^	<0,001	2,10; 0,20 (2,00–2,20)^b^	0,003	0,349 (U de Mann-Whitney)	–
Vitamina D, nmol/L	59,8 ± 18,7 (55,0–64,6)^a^	0,185	65,1 ± 20,1 (59,1–71,1)^a^	0,052	0,165 (t-Student)	0,593
IGFBP-3, μg/mL	5,69 ± 1,27 (5,37–6,02)^a^	0,200	5,22 ± 0,95 (4,93–5,51)^a^	0,200	**0,042** (t-Student)	0,061
IGF-I, ng/mL	208,0; 109 (162,5–271,5)^b^	<0,001	216,0; 111 (159,0–270,0)^b^	0,007	0,866 (U de Mann-Whitney)	–

^a^Media ± desviación estándar (IC 95 %); ^b^mediana; rango intercuartílico (Q1-Q3); ^c^Kolmogorov-Smirnov con modificación de Lillefors. Los valores en negrita hacen referencia a los parámetros donde las pruebas de significación estadística mostraron una diferencia estadísticamente significativa entre los dos grupos para un nivel de confianza del 95 %.

Se encontraron diferencias entre el grupo de niños con sobrepeso/obesidad y el grupo de niños normopeso, en la concentración de fósforo y en la de IGFBP-3, siendo, más elevada, en ambos casos, de forma estadísticamente significativa, en el grupo de niños con sobrepeso/obesidad, (p<0,042) y (p<0,042), respectivamente. En el resto de parámetros, a pesar de que la BAP, la osteocalcina, el magnesio, la vitamina D e IGF-I eran inferiores en el grupo de los niños obesos y el calcio era superior, tras la aplicación de los diferentes tests estadísticos, resultó que las concentraciones eran comparables en ambos grupos (normopeso y sobrepeso/obesidad) para un nivel de confianza del 95 %.

Cuando diferenciamos por sexo, en el grupo de niños/as normopeso, hubo diferencias, estadísticamente significativas en la concentración de BAP y en la de IGF-1, encontrando valores superiores, en ambos casos, en las niñas, p<0,05.

En el grupo de niños/as con sobrepeso/obesidad encontramos diferencias, estadísticamente significativas en la concentración de IGFBP-3, la cual fue también superior en el grupo de las niñas, p=0,026.

Cuando comparamos los niños normopeso, frente a niños con sobrepeso/obesidad, obtuvimos diferencias, estadísticamente significativas en la concentración de fósforo, la cual fue superior en el grupo de los niños sobrepeso, p=0,016, no habiendo obtenido diferencias estadísticamente significativas, en las niñas.

**Figura 2:**
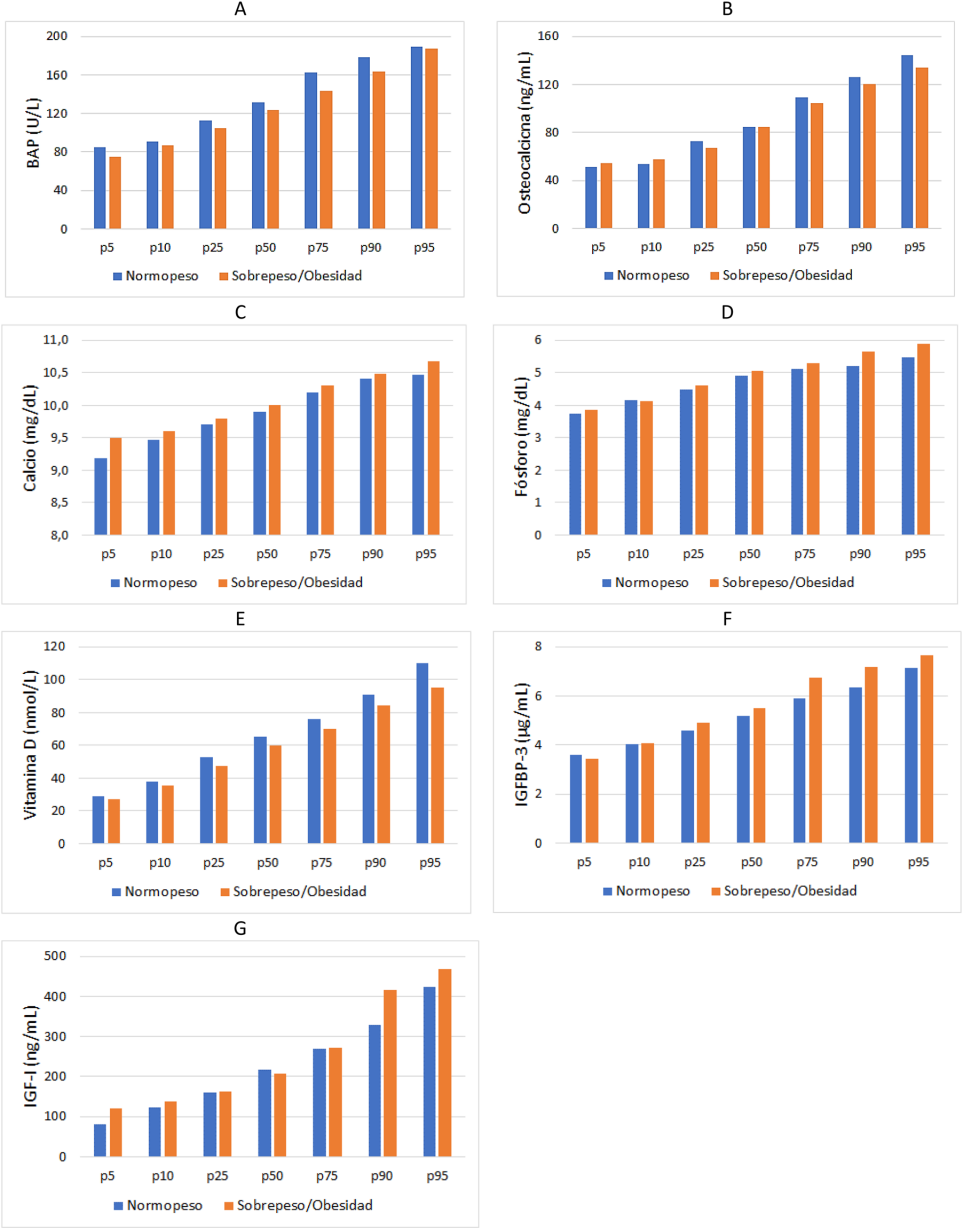
Percentiles de los parámetros óseos en niños con normopeso y niños con sobrepeso/obesidad. (A) BAP, (B) osteocalcina, (C) calcio, (D) fósforo, (E) vitamina D, (F) IGFBP-3, (G) IGF-I.

En la Figura 2 se muestran las diferencias existentes entre los distintos percentiles, obtenidas para los parámetros estudiados, en cada uno de los grupos.

La correlación entre los distintos parámetros bioquímicos y el IMC, edad y sexo se muestran en la Tabla 3. Respecto a la correlación con el IMC, existía correlación negativa con BAP y positiva con IGFBP-3 y respecto a la edad, mostraron correlación positiva BAP y osteocalcina. En relación al sexo, existía correlación positiva en las niñas con BAP, IGFBP-3 y con IGF-I.

**Tabla 3:** Correlación de los parámetros bioquímicos con edad, sexo e Índice de masa corporal.

	Significación test de normalidad^a^	Correlación con IMC		Correlación con edad		Correlación con sexo^b^	
Parámetro		Coeficiente correlación r	Significación estadística	Coeficiente correlación r	Significación estadística	Coeficiente correlación r	Significación estadística
Edad	0,200	–	–	–	–	–	–
IMC	<0,001	–	–	–	–	–	–
BAP	0,200	**−0,193**	**0,049**	**0,235**	**0,016**	**0,184**	**0,022**
Osteocalcina	0,002	−0,011	0,911	**0,247**	**0,011**	0,137	0,090
Calcio	0,142	0,115	0,240	0,030	0,759	0,013	0.872
Fosforo	0,106	0,146	0,135	−0,094	0,340	0,056	0,495
Magnesio	<0,001	−0,136	0,166	−0,043	0,659	−0,021	0,814
Vitamina D	0,060	−0,130	0,184	−0,095	0,333	0,060	0.455
IGFBP-3	0,200	**0,276**	**0,005**	0,169	0,086	**0,241**	**0,003**
IGF-I	<0,001	0,079	0,428	0,384	<0,001	**0,189**	**0,019**

^a^Test de normalidad de Kolmogorov-Smirnov con la modificación de Lilliefors; ^b^si la correlación es positiva significa que en las niñas el valor del parámetro es mayor que en los niños y si es negativa es mayor en los niños. Los valores en negrita representan aquellos parámetros donde el coeficiente de correlación es estadísticamente significativo para un nivel de confianza del 95 %.

**Figura 3:**
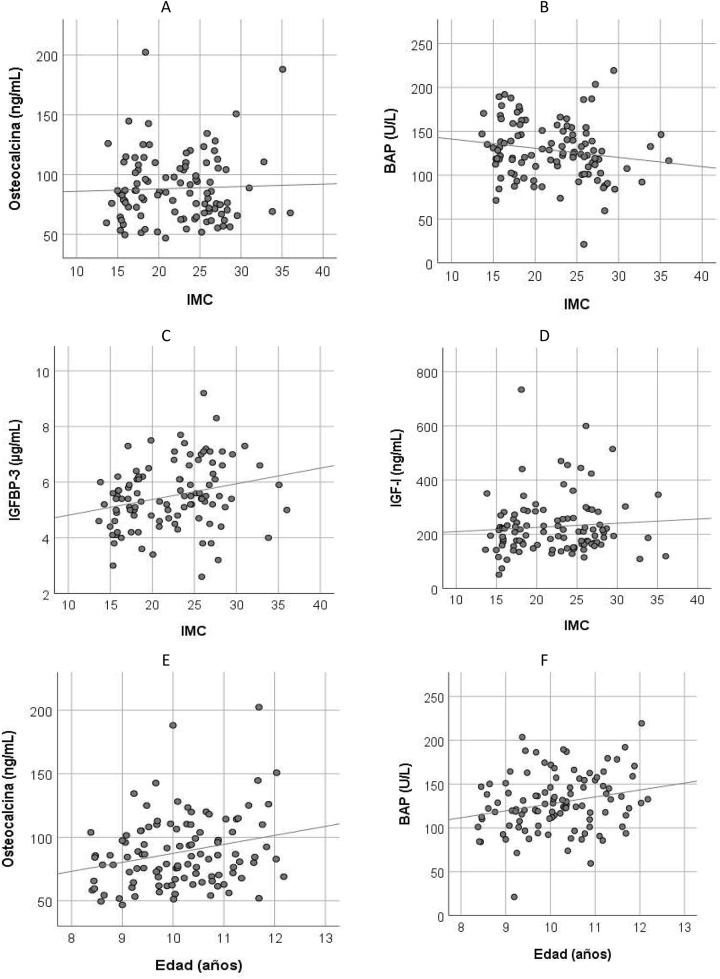
Correlación de los parámetros del metabolismo óseo e IGFBP-3 con IMC y con la edad. (A) Osteocalcina, (B) BAP, (C) IGFBP-3, (D) IGF-I, todos ellos respecto a IMC, (E) osteocalcina y (F) BAP, ambas respecto a la edad.

La representación gráfica de la correlación existente entre BAP, Osteocalcina, IGFBP-3 e IGF-I con el IMC, así como entre osteocalcina y BAP respecto a la edad se muestran en la Figura 3.

## Discusión

Aparte de la importancia que tiene en los niños alcanzar una adecuada masa ósea para prevenir la osteoporosis en la edad adulta, existen diferentes estudios prospectivos que relacionan el aumento del contenido en grasa de los músculos con una disminución de la funcionalidad de los mismos, lo cual se va a traducir en un efecto perjudicial sobre el crecimiento de los niños, contribuyendo todo ello, al gran interés que presenta conocer en qué medida afecta la obesidad al metabolismo óseo de estos niños.

Pese a que existen diferentes estudios que demuestran que los niños con sobrepeso/obesidad tienen un mayor contenido mineral óseo que los niños normopeso, algunos autores han encontrado un aumento en el riesgo de fracturas en extremidades en estos niños, lo cual sugiere que presentan huesos de menor calidad ósea [18].

En los niños con sobrepeso/obesidad la calidad ósea está influenciada tanto por la liberación de citoquinas inmunomoduladoras inflamatorias, como por la sobrecarga mecánica que experimenta el hueso, ya que tanto el tejido adiposo como el óseo son tejidos metabólicamente activos, con una clara interrelación entre ambos [19]. Asimismo, existen evidencias experimentales en modelos animales que ponen de manifiesto que la sustitución de médula ósea por tejido adiposo, promueve una inflamación de bajo grado, reduciendo la actividad osteoblástica y promoviendo la actividad osteoclástica.

En nuestro estudio se utilizaron como marcadores de formación ósea BAP y osteocalcina, que son algunos de los más utilizados, y, al igual que en el estudio de Saber y cols. [15], realizado en un grupo de niños de 7,55 ± 3,34 años, hemos encontrado concentraciones similares de osteocalcina en los niños con sobrepeso/obesidad, frente a los niños normopeso, así como mayor concentración de fósforo en el grupo de niños con sobrepeso/obesidad. Asimismo, en el estudio de Mosca y cols [20]. tampoco se encontraron diferencias estadísticamente significativas al comparar la concentración de osteocalcina entre niños normopeso y niños con sobrepeso/obesidad de edades comprendidas entre 10 y 13 años.

Sin embargo, algunos otros autores han encontrado menor concentración de los marcadores óseos en los niños con sobrepeso/obesidad frente a los niños normopeso [13, 14] e incluso otros encontraron concentraciones más elevadas en los niños obesos al compararlas con las obtenidas en los niños normopeso [21].

No obstante, en nuestro estudio, aunque únicamente hemos encontrado diferencias estadísticamente significativas para la concentración de fósforo e IGFBP-3, la cual fue más elevada, en el grupo de niños con sobrepeso/obesidad, las concentraciones de BAP, osteocalcina, magnesio, vitamina D e IGF-I fueron inferiores en el grupo de niños con sobrepeso/obesidad, sin llegar a alcanzar las diferencias significación estadística.

En cuanto a la concentración de fósforo, además de estar más elevada en el grupo de niños con sobrepeso/obesidad, respecto a los niños normopeso (p<0,042), aproximadamente el 10 % de los niños con sobrepeso/obesidad tendrían valores por encima del límite superior del rango de referencia (3,7–5,6 mg/dL, para la edad de 4–11 años) [22], mientras que en el grupo de niños normopeso, todos presentaban concentraciones dentro del rango de referencia.

El hecho de que los niños con sobrepeso/obesidad, de 8–12 años, presenten niveles más elevados de fósforo e IGFBP-3, hace pensar en que existe un crecimiento mayor en estos niños, puesto que, además, la talla de los niños con sobrepeso/obesidad era mayor que la de los niños normopeso.

En la concentración de calcio y vitamina D, parámetros relacionados con la concentración de fósforo [23], sin embargo, no llegamos a encontrar diferencias significativas entre los dos grupos.

En cuanto a la concentración de calcio, existen resultados algo contradictorios, según los estudios consultados, encontrando en algunos de ellos, concentración más elevada en los niños obesos [24], mientras que en otros no se encontraron diferencias significativas [25].

La concentración de vitamina D fue menor, en ambos estudios [24] y [25] en los niños obesos, al compararla con la que presentaban los niños normopeso. Además, en un metaanálisis llevado a cabo en el año 2020 se encontró que el riesgo relativo de asociación entre obesidad y déficit de vitamina D era de 1,41 (95 % CI: 1,26–1,59) [26], lo cual sugiere que los niños y adolescentes con sobrepeso u obesidad tienen un mayor riesgo de deficiencia de vitamina D. En nuestro estudio, aunque no se llegaron a encontrar diferencias significativas, entre ambos grupos, en la concentración de vitamina D, en todos los percentiles existe un ligero aumento de la concentración de vitamina D en los niños normopeso, respecto a la de los niños con sobrepeso/obesidad, según puede apreciarse en la Figura 3. Respecto a la relación existente entre los marcadores óseos y el IMC, la mayoría de estudios no encuentran correlación entre osteocalcina e IMC [15, 27] en coincidencia con nuestros resultados y en cuanto a la correlación entre BAP e IMC, Cao y cols [28]. en el año 2022 encontraron correlación negativa, en concordancia, asimismo, con nuestros resultados.

Respecto a los factores de crecimiento, la bibliografía existente en torno a la asociación que presentan con el metabolismo óseo en los niños con sobrepeso/obesidad es escasa y en algunos casos también contradictoria. Por una parte, en el estudio de Gajewska y cols., llevado a cabo en 2015 [29], encontraron que IGF-I era superior en niños con obesidad frente a los niños normopeso e IGFBP-3 total era similar en ambos grupos, mientras que, en otro estudio, realizado en el año 2021 por Czogala y cols. [30], se observaron concentraciones similares de IGF-I, en ambos grupos y la concentración de IGFBP-3 era superior en el grupo de niños con obesidad, resultados, estos últimos, acordes con los nuestros.

Como se habrá podido observar, son muy pocos los estudios sobre marcadores óseos en niños con normopeso y con sobrepeso/obesidad, por lo que sería de gran interés realizar más estudios sobre este interesante tema, ya que algunos de los resultados encontrados en la literatura y en nuestro propio estudio son bastante controvertidos. Incluso serían necesarios estudios longitudinales para llegar a identificar los cambios en las conexiones existentes entre los marcadores óseos y el tejido adiposo, cuando se produce el cambio de peso normal a obesidad y cuáles son las causas que producen la alteración en las hormonas que regulan estos procesos.

Al parecer, las hormonas calciotrópicas, que regulan de forma muy fina el metabolismo mineral están influenciadas por factores hormonales, directamente involucrados en el metabolismo energético, existiendo bastante evidencia sobre la interrelación existente entre la leptina, las hormonas calciotrópicas y los marcadores de recambio óseo, lo cual demuestra el importante papel del tejido graso sobre el esqueleto y el metabolismo óseo.

Por todo ello, una cuestión de interés sería poder ampliar nuestro tamaño muestral, pudiendo incluso llegar a establecer una diferenciación entre niños/as con sobrepeso y niños/as con obesidad, así como estudiar una serie de hormonas y de algunos parámetros que se ha observado pueden tener relación de interés con la obesidad y su influencia en el metabolismo óseo, como la leptina, la hormona paratiroidea (PTH) y los marcadores óseos de resorción (β-CTX o ICTP), entre otros.

En conclusión, hemos encontrado concentraciones más elevadas tanto de fósforo como de IGFBP-3 en los niños con sobrepeso/obesidad, con respecto a los niños normopeso, lo que evidencia un crecimiento mayor en estos niños, y aunque no se llegó a alcanzar significación estadística, la concentración de BAP y osteocalcina fue inferior a la de los niños normopeso, lo cual unido a la correlación negativa entre BAP e IMC encontrada, puede indicar que ya en edades tan tempranas el sobrepeso/obesidad puede afectar a la salud ósea.

## Supplementary Material

Supplementary Material

## Material Suplementario

Este artículo incluye material suplementario (https://doi.org/10.1515/almed-2023-0065).

## References

[1] De Leonibus C, Marcovecchio ML, Chiavaroli V, de Giorgis T, Chiarelli F, Mohn A (2014). Timing of puberty and physical growth in obese children: a longitudinal study in boys and girls. Pediatr Obes.

[2] Blundell JE, Baker JL, Boyland E, Blaak E, Charzewska J, de Henauw S (2017). Variations in the prevalence of obesity among European countries, and a consideration of possible causes. Obes Facts.

[3] Lavie CJ, Milani RV, Ventura HO (2009). Obesity and cardiovascular disease. J Am Coll Cardiol.

[4] AESAN Estudio ALDINO 2019 2020.

[5] de Bont J, Bennett M, León-Muñoz LM, Duarte-Salles T (2022). Prevalencia e incidencia de sobrepeso y obesidad en 2,5 millones de niños y adolescentes en España. Rev Esp Cardiol.

[6] de Ruiter I, Olmedo-Requena R, Sánchez-Cruz JJ, Jiménez-Moleón JJ (2017). Tendencia de la obesidad infantil y el bajo peso por año de nacimiento y edad en España, 1983-2011. Rev Esp Cardiol.

[7] Jürimäe J (2010). Interpretation and application of bone turnover markers in children and adolescents. Curr Opin Pediatr.

[8] Boot AM, de Ridder MAJ, van der Sluis IM, van Slobbe I, Krenning EP, de Muinck Keizer-Schrama SMPF (2010). Peak bone mineral density, lean body mass and fractures. Bone.

[9] Lupsa BC, Insogna K (2015). Bone health and osteoporosis. Endocrinol Metab Clin North Am.

[10] Khadilkar A, Chiplonkar S, Agrawal DP, Sanwalka N, Khadilkar V (2016). Bone health status in Indian overweight/obese children. Indian J Pediatr.

[11] van Leeuwen J, Koes BW, Paulis WD, van Middelkoop M (2017). Differences in bone mineral density between normal-weight children and children with overweight and obesity: a systematic review and meta-analysis. Obes Rev.

[12] Rauchenzauner M, Schmid A, Heinz-Erian P, Kapelari K, Falkensammer G, Griesmacher A (2007). Sex- and age-specific reference curves for serum markers of bone turnover in healthy children from 2 Months to 18 years. J Clin Endocrinol Metab.

[13] Geserick M, Vogel M, Eckelt F, Schlingmann M, Hiemisch A, Baber R (2020). Children and adolescents with obesity have reduced serum bone turnover markers and 25-hydroxyvitamin D but increased parathyroid hormone concentrations – results derived from new pediatric reference ranges. Bone.

[14] Bini V, Igli Baroncelli G, Papi F, Celi F, Saggese G, Falorni A (2004). Relationships of serum leptin levels with biochemical markers of bone turnover and with growth factors in normal weight and overweight children. Horm Res Paediatr.

[15] Saber LM, Mahran HNF, Baghdadi HH, Al Hawsawi ZMH (2015). Interrelationship between bone turnover markers, calciotropic hormones and leptin in obese Saudi children. Eur Rev Med Pharmacol Sci.

[16] Mengel E, Tillmann V, Remmel L, Kool P, Purge P, Lätt E (2018). The associations between the changes in serum inflammatory markers and bone mineral accrual in boys with overweight and obesity during pubertal maturation: a 3-year longitudinal study in Estonian boys. Osteoporosis Int.

[17] Cole TJ, Lobstein T (2012). Extended international (IOTF) body mass index cut-offs for thinness, overweight and obesity. Pediatr Obes.

[18] Kessler J, Koebnick C, Smith N, Adams A (2013). Childhood obesity is associated with increased risk of most lower extremity fractures. Clin Orthop Relat Res.

[19] da Silva SV, Renovato‐Martins M, Ribeiro‐Pereira C, Citelli M, Barja‐Fidalgo C (2016). Obesity modifies bone marrow microenvironment and directs bone marrow mesenchymal cells to adipogenesis. Obesity.

[20] Mosca LN, Goldberg TBL, da Silva VN, Kurokawa CS, Rizzo ACB, da Silva CC (2017). The impact of excess body fat on bone remodeling in adolescents. Osteoporosis Int.

[21] Radetti G, Franceschi R, Adami S, Longhi S, Rossini M, Gatti D (2014). Higher circulating parathormone is associated with smaller and weaker bones in obese children. Calcif Tissue Int.

[22] Lockitch G, Halstead AC, Albersheim S, MacCallum C, Quigley G (1988). Age- and sex-specific pediatric reference intervals for biochemistry analytes as measured with the Ektachem-700 analyzer. Clin Chem.

[23] Allgrove J (2015). Physiology of calcium, phosphate, magnesium and vitamin D. Endocr Dev.

[24] Plesner JL, Dahl M, Fonvig CE, Nielsen TRH, Kloppenborg JT, Pedersen O (2018). Obesity is associated with Vitamin D deficiency in Danish children and adolescents. J Pediatr Endocrinol Metabol.

[25] Reinehr T, de Sousa G, Alexy U, Kersting M, Andler W (2007). Vitamin D status and parathyroid hormone in obese children before and after weight loss. Eur J Endocrinol.

[26] Fiamenghi VI, Mello EDD (2021). Vitamin D deficiency in children and adolescents with obesity: a meta-analysis. J Pediatr.

[27] Zhang J, Zhou W, Zhang Y, Liu C, Yu F, Jiang Y (2023). Relationship between body mass index and bone turnover markers in girls with idiopathic central precocious puberty. Int J Clin Pract.

[28] Cao B, Liu M, Luo Q, Wang Q, Liu M, Liang X (2022). The effect of BMI, age, gender, and pubertal stage on bone turnover markers in Chinese children and adolescents. Front Endocrinol.

[29] Gajewska J, Klemarczyk W, Ambroszkiewicz J, Szamotulska K, Chełchowska M, Weker H (2015). Associations between IGF-I, IGF-binding proteins and bone turnover markers in prepubertal obese children. J Pediatr Endocrinol Metabol.

[30] Czogała W, Strojny W, Tomasik P, Multanowski MB, Wójcik M, Miklusiak K (2021). The insight into insulin-like growth factors and insulin-like growth-factor-binding proteins and metabolic profile in pediatric obesity. Nutrients.

