# Supplementary material for: Metabolismo óseo en niños aragoneses con normopeso y niños con sobrepeso/obesidad
Source: Adv Lab Med. 2024 Jan 1;5(1):75–84. [Article in Spanish] doi: 10.1515/almed-2023-0065 (PMC11019878; doi:10.1515/almed-2023-0065)
Supplement: Supplementary file 1 — Supplementary Material [file j_almed-2023-0065_suppl_001.docx]

**Encuesta realizada a los niños del estudio**

- **EDAD**:

- **SEXO**:

- **MES EXTRACCIÓN ANALÍTICA (ESTACIÓN):**

**- PESO/Z-SCORE: IMC/ Z-SCORE:**

**- TALLA/ Z-SCORE:**

- **PATOLOGÍAS PREVIAS**, especialmente las relacionadas con el metabolismo óseo:

o Fracturas. ¿Cuantas? Localización.

o Tratamiento de base: anticonvulsivantes, glucocorticoides

o Infecciones.

o Enfermedades relacionadas con el sistema inmunitario.

o Alteraciones concentración glucosa, diabetes mellitus en el niño.

- **HISTORIA FAMILIAR** (principalmente enfermedades óseas, enfermedad

cardiovascular o enfermedades metabólicas)

- **PROFILAXIS:**

o ¿Realiza profilaxis con vitamina D? SI NO
